# Paratuberculosis Vaccination Causes Only Limited Cross-Reactivity in the Skin Test for Diagnosis of Bovine Tuberculosis

**DOI:** 10.1371/journal.pone.0080985

**Published:** 2013-11-26

**Authors:** Joseba M. Garrido, Patricia Vazquez, Elena Molina, Jose M. Plazaola, Iker A. Sevilla, Maria V. Geijo, Marta Alonso-Hearn, Ramon A. Juste

**Affiliations:** 1 NEIKER-Tecnalia, Animal Health Department, Derio, Bizkaia, Spain; 2 Diputación Foral de Gipuzkoa, Donostia-San Sebastián, Gipuzkoa, Spain; Texas A&M Health Science Center, United States of America

## Abstract

Although there is a wide consensus on the efficacy of paratuberculosis vaccination to limit economic losses, its use has been restricted because of its interference in the diagnosis of tuberculosis. Data from a vaccine clinical trial in the Basque Country (Spain) has been evaluated in relationship with bovine tuberculosis intradermal test results. The trial included two herds applying a Test and Culling strategy and five applying an inactivated vaccine. The vaccine was applied to animals of all ages present in each vaccinated herd when joining the trial, and then to all the replacers within their first three months of life. Yearly testing done with the comparative intradermal test (CIT) was applied to all animals older than 6 weeks. Between 2005 and 2011, the study generated 2,033 records from Vaccinated Herds (VH) and 2,252 from Test and Cull herds (TC). Pre-vaccination positive results rate was 2.40% among the 7 herds in the single bovine intradermal tuberculin test (BSIT). Two years later it rose to 20.42% in the VH and remained below at 0.75% in the TC. Applying the CIT reduced these rates to only 0.58% in the VH and to 0.25% in the TC ons. Regarding time since each animal joined the program, the proportion of positives to BSIT was variable and, in some cases, significantly different between time points. With regard to the age of vaccination, no significant differences were found between vaccination within the first year of life and afterwards. Vaccinated animals showed seventeen times more reactions than the non-vaccinated in the BSIT, but only four times more in the CIT. In conclusion, comparative intradermal test can be a useful tool to differentiate paratuberculosis vaccine cross-reactions from specific bovine tuberculosis reactions according to the European and Spanish legislation.

## Introduction

Paratuberculosis or Johne's disease is a chronic granulomatous inflammation that mainly affects ruminants and that is generally accepted to be caused by *Mycobacterium avium* subspecies *paratuberculosis* (MAP). Paratuberculosis is one of the diseases with the larger disruption effects on current cattle production systems, especially in dairy herds [1]. Factors such as fecal-oral route of transmission, high resistance of mycobacteria in the environment, and long incubation period make it difficult to control this disease.

Strategies for paratuberculosis control can be classified into two fundamental types: testing and culling (TC) and vaccination. Although TC with different tests and variable degrees of management changes are the most favored strategies by veterinary consultants, and despite having been successful at the farm level, these strategies have not met generalized acceptance for use at a wider scale. The main cause is that current diagnostic techniques are of a very limited sensitivity during the early phases of the infection and, as a consequence, tend to leave undetected many infected animals. This, together with the ubiquity of the agent and the high economic costs and lack of sustainability of repeated testing and continuous culling of animals at the beginning of their productive life, makes eradication by this strategy an expensive and ultimately unrealistic goal. In contrast, vaccination has been longtime used both in the bovine and in the ovine species with good results when issues such as: a) reduction of clinical cases and excretion rates, b) positive shift in the type of lesions, or c) the cost-benefit ratio in reference to TC strategy have been used as the outcome variables [2], [3], [4], [5], [6], [7], [8]. However, there are two main issues that have limited a wider use of vaccination. The first is the already mentioned misconception of setting immediate eradication as the main goal, instead of focusing just on disease control by quick reduction of economic losses and MAP environmental burden on farms until reaching near-eradication levels. The second issue is the interference of vaccination with the diagnosis of tuberculosis (TB) which makes Animal Health Authorities reluctant to allow its use in the context of bovine tuberculosis national eradication programs [9], [10], in spite of authorizing its use in small ruminants not submitted to TB programs.

Nearly complete eradication of bovine tuberculosis in the Basque Country, as well as an increasing prevalence of clinical cases of paratuberculosis, led the local Animal Health Authorities to support a vaccination trial designed to test the efficacy of an inactivated MAP vaccine (Silirum®, CZV, Porriño, Spain) in dairy cows exposed to MAP. Since field information on paratuberculosis vaccination and intradermal tests results interference is scarce and old, we thought it was important to use the information generated in this trial to investigate the degree in which vaccinated cows can become false positives in the bovine single (BSIT) and comparative intradermal test (CIT) and how it compares with natural infection interference in TC strategies.

## Materials and Methods

### Ethics statement

Animals used in this study belong to commercial farms and, towards collection of the data for this study, have been submitted only to the standard clinical practices specifically regulated by the European and Spanish legislation on tuberculosis and brucellosis control programs, plus fecal sampling.

### Herds selection

Seven bovine herds located in the Basque Country with a history of clinical paratuberculosis were selected. Six of the herds were dairy farms and one was beef. Serologic and microbiologic prevalence of MAP infection in these herds ranged between 2% and 10%. Requirements for joining the trial were that the herd had been tuberculosis officially free for, at least, ten years before joining the trial and that the owners signed an agreement to facilitate information on any management aspect related to the trial, to collaborate in the testing and sampling and to refrain from selling breeding animals to other farms.

Two herds, each with an average of 220 animals at each yearly testing, applied a TC strategy and the other five, with an average of 100 animals, a vaccination one. The beef herd was included in this last group. All the dairy herds followed the same intensive management, while the beef herd followed a semi-extensive system. Vaccinated herds (VH) joined the program between July 2005 and April 2006, and both TC did it in June 2006. Data from annual samplings have been collected yearly until 2011.

### Study design

The CIT involves the separate intradermal injection of purified protein derivatives (PPD) from *Mycobacterium bovis* and from *Mycobacterium avium*, and the subsequent detection of swelling and induration with or without any other regional signs at the injection sites. The relative change in skin thickness at the two injection sites is used to differentiate *M. bovis* infection reaction from infection or sensitizing contact with non-tuberculous mycobacteria.

Vaccinated and non-vaccinated calves older than 6 weeks were yearly tested throughout the study at the time of joining the program (M0) and at 12 (M12), 24 (M24), 36 (M36), 48 (M48) and 60 (M60) months. The CIT was carried out by the Official Veterinary Services according to the relevant European Directive (EU Council Directive 64/432/CEE and RD 2611/1996). Briefly, two sites of the mid-neck were shaved and inoculated with 0.1 mL of bovine PPD in one site (2,500 UI, CZ Veterinaria, S.L.; Porriño, Spain) and with 0.1 mL of avian PPD in the same volume in another one (2,500 UI, CZ Veterinaria) separated about 10 cm from the other place. After 72 h, the skin fold thickness at the injection sites were measured with a caliper. Only standard interpretation was used because of the low TB prevalence status of the Basque Country. Actually, since freedom of TB was a condition for joining the program and some degree of interference was already expected because of the previous natural paratuberculosis infection status of all the farms, it would make no sense to use a more strict interpretation. This fact alone, indeed, will interfere with an accurate assessment of the TB status of the animals after the addition of an artificial mycobacterial immunization. Therefore, animals were considered positive to the CIT if showing a skin fold thickness increase greater than 4 mm in the bovine PPD inoculation site than in the avian inoculation site.

Vaccination was carried out as described before [5]. Briefly, one mL of Silirum® MAP vaccine (CZ Veterinaria, S.L.; Porriño, Spain) was subcutaneously administered with a 0.9×25 mm needle into the dewlap of all the animals at the moment of joining the trial, and then in subsequent years to all new born female calves older than 1 month and younger than 3 months and intended for replacement. Each dose contained 2.5 mg of the heat-killed 316F strain of MAP in an oil adjuvant. Animals from unvaccinated herds were considered as control and submitted to Testing and culling by ELISA and fecal PCR on an annual schedule. Only positive animals to both ELISA and fecal PCR were culled.

### Slaughterhouse follow-up

Standard yearly culling rate amounted to approximately 25% of the animals of each herd. These animals were sent to the slaughterhouse where they were submitted to the compulsory ante- and post-mortem inspection by Official Public Health Veterinarians.

### Data analysis

A total of 4,285 records, 2,033 of them from VH and 2,252 from TC were obtained. These records were scored as 0 or 1 if they had a negative or a positive result, respectively, in each of two dependent variables: BSIT and CIT. These binomial variables were treated as quantitative variables where the mean was the frequency of each possible outcome in order to submit them to the Proc GLM of the SAS statistical package (SAS Institute, Cary, NC, USA) for analysis of variance of main effects and least square means comparison with the Student-t test with the Tukey-Kramer adjustment for multiple comparisons at each post-intervention time. The control level was the pre-vaccination or TC group mean/frequency. Independent variables were vaccination (yes or not) and one of two time variables: natural year of testing since the beginning of the follow-up and individual post-vaccination year. Both farm and individual animal were considered random effects that were not relevant for statistical inference of vaccination interference and whose effects were left as part of the error in the general linear model. For the herd reference, time was counted starting at the first intervention: pre-vaccination [M0] and then each natural year post-vaccination [M12, M24, M36, M48 and M60]. For individual reference, time was counted since vaccination (VH) or first testing (TC) and then categorized in one-year periods (pre-vaccination [00A], 0–12 months post-vaccination [01A], 12–24 months post-vaccination [02A], 24–36 months [03A], 36–48 months [04A] and 48–60 months [05A]). Additionally, age at vaccination was considered as a possible factor for persistence of strong immune responses and therefore was used in the models with two levels: <12 months and > = 12 months.

## Results

### General results (Herd reference)

When all records were analyzed together regardless of time since vaccination and age at vaccination, the model with strategy, age at vaccination and yearly testing showed that that 9.42% of the animals from the VH herds were positive in the BSIT versus only 0.56% from the TC herds (least-square means; p<0.0001). Applying the same analysis to the CIT readings, showed that 0.10% of the vaccinated animals were positive, against 0.03% in the TC group (p = 0.2942).

**Figure 1:**
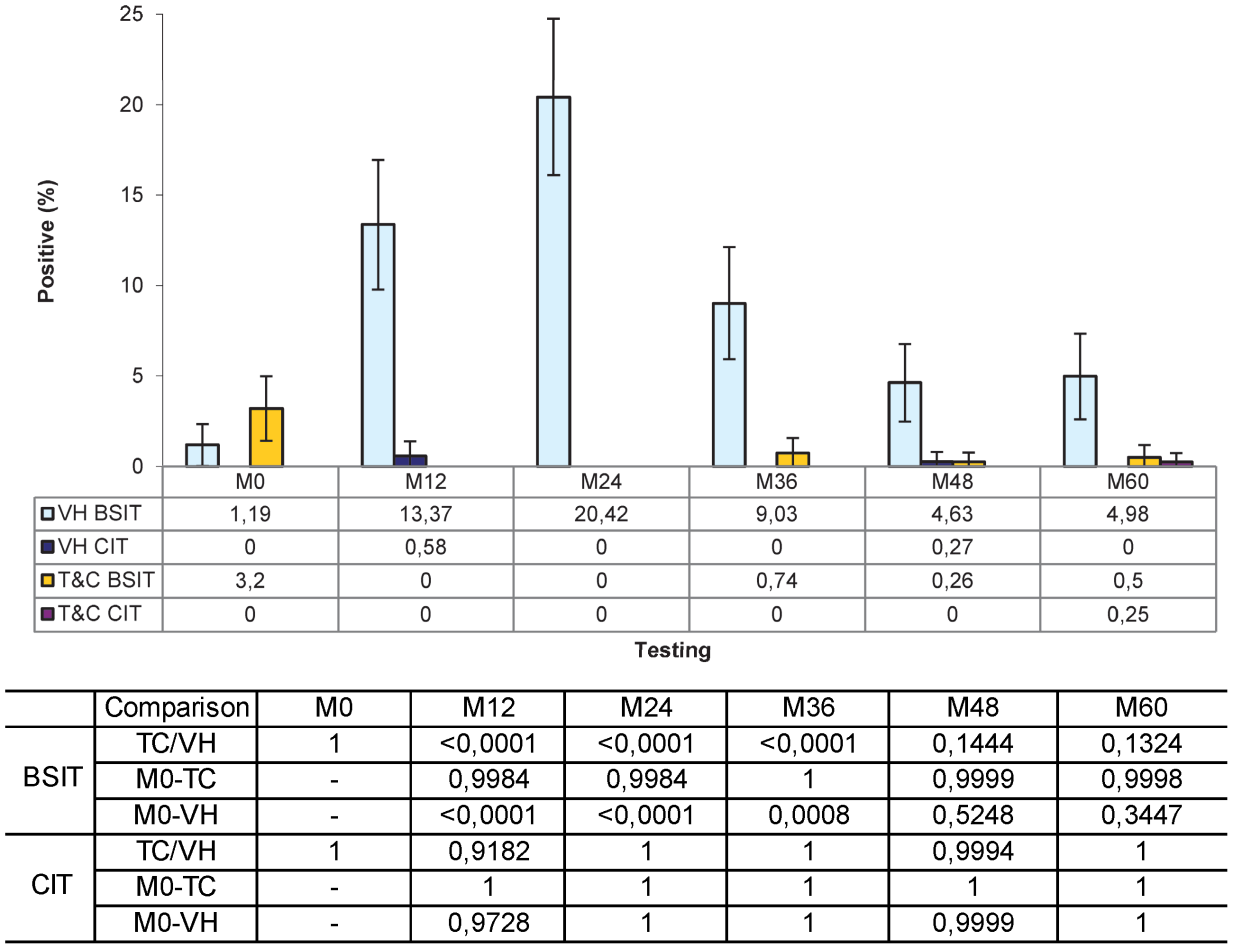
Percentage of positive animals in vaccinated (VH) and Testing and Culling (TC) herds to bovine single (BSIT) and comparative (CIT) intradermal tests throughout the study. No significant difference (p = 1.0000) between strategies was observed in the BSIT at the initial testing (M0), nor at the last two testings (p = 0.1444 and p = 0.1324); but the frequencies were significantly different at each of the three immediate post-vaccination testings (p<0.0001). Regarding evolution along time, only frequencies in the M12, M24 and M36 yearly testings were significantly different (p<0,0009) from M0 in VH. No statistically significant differences were observed between strategies, or between testings within each strategy in the CIT. Table on the x-axis shows frequencies and table below axis legend shows statistical probabilities for the differences. These p values were obtained with a Student t-test with the Tukey-Kramer adjustment for multiple comparisons on the least square frequency means calculated in the analysis of variance.

**Figure 2:**
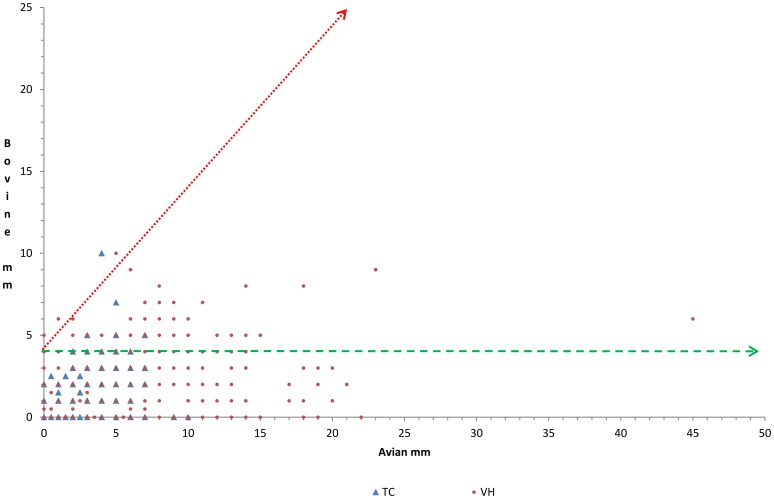
Skin–fold thickness increases at 72 h after PPD-Av and PPD-Bov intradermal inoculation in vaccinated (VH) an Testing and culling (TC) herds at all the yearly testings. A positive bovine reaction, which was 4(0.05%) corresponded to TC herds and three (0.14%) occurred in VH herds.

In the same model, the percentage of positivity to BSIT in vaccinated farms ranged from 20.78% at the M24 testing to 5.04% at the M48 which compared to the M0 represented significant differences at M12 (p<0.0001), M24 (p<0.0001) and M36 (p = 0.0008), but not at M48 (p = 0.5248) and M60 (p = 0.3447). In the TC herds, these rates never exceeded 0.74% (M36) with p values always over 0.9980. Regarding the CIT, positive reactions were only observed at M12 and M48 testing, with 2 (0.41%) and 1 (0.21%) positive animals in the VH group, respectively, and at the M60 testing of the TC with 1 (0.15%) positive animal (Figure 1) always with p values close to 1.0000. Comparing the frequency of positives in each type of interpretation, no significant difference (p = 1.0000) between both strategies (0.69% VH vs. 1.86% TC) was observed in the BSIT at the initial testing. However, in the subsequent testings, significant differences (p<0.0001) were observed at M12 (14.89% VH vs. 0.00% TC), M24 (20.78% VH vs. 0.00% TC) and M36 (9.19% VH vs. 0.72% TC), but not at M48 (5.04% VH vs. 0.45 TC; p = 0.1444) and M60 (5.99% VH vs. 0.30% TC; p = 0.1324). No significant differences at all were observed when using the CIT interpretation (Figure 1). Individual skin-fold thickness increase at 72 h in the PPD-Av and PPD-Bov intradermal inoculation sites in VH and TC herds are shown in Figure 2. Only four tests from four different animals yielded a bovine positive result. One (0.25% of that testing ) corresponded to a TC herd in its 5 years follow-up that had had five previous fully negative results. The other three (0.58% and 0.27% of their respective testings) occurred in 2 vaccinated animals at 1 year post-vaccination (adult vaccination) and in another one at 4 years post-vaccination (calf vaccination). One of the former was culled after its positive test at the first year post-vaccination. The other three had one subsequent negative test. The cow vaccinated as a calf was still alive in the last herd control. The other two were killed and had no TB lesions reported.

### Results according the time elapsed since vaccination (Individual reference)

**Figure 3:**
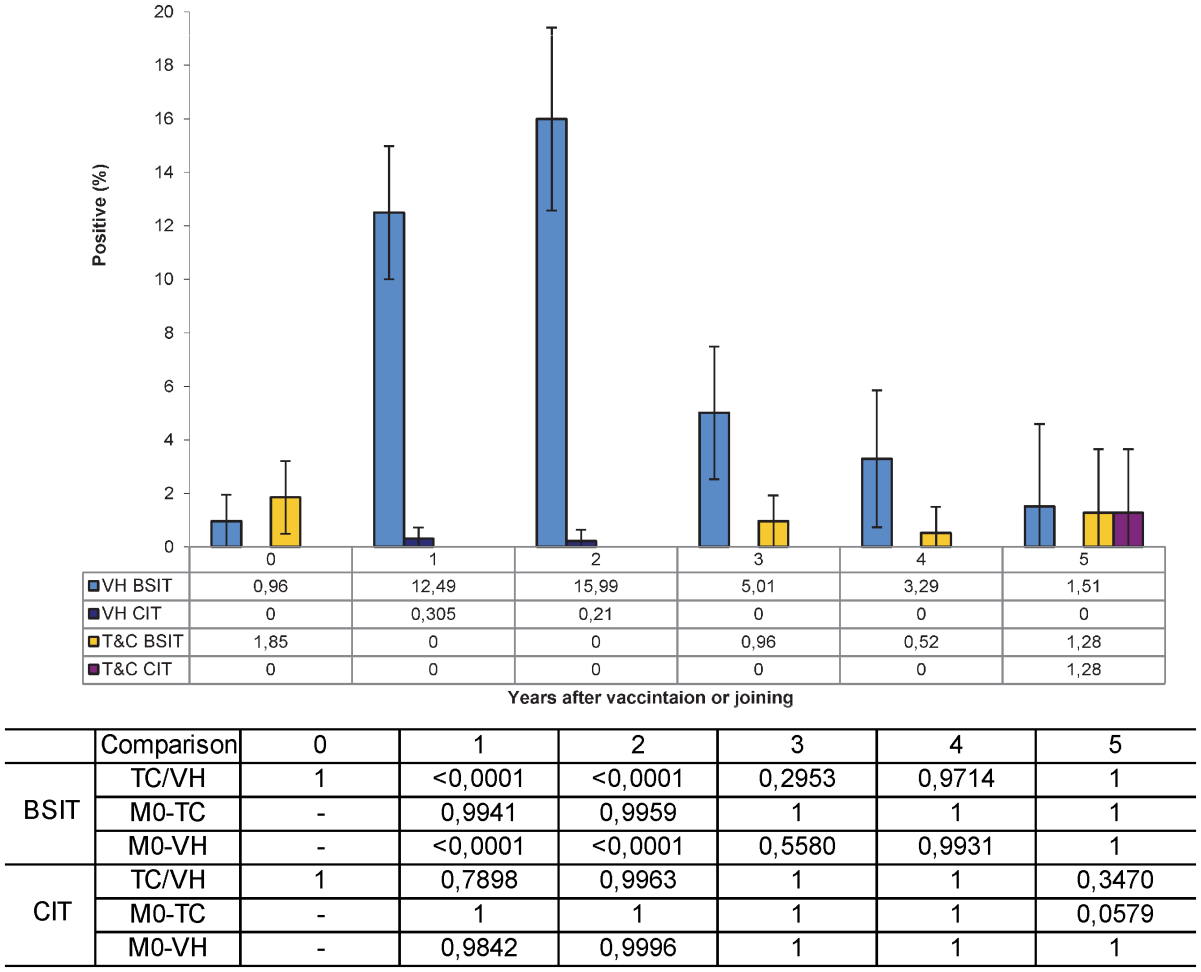
Percentage of positive animals to bovine single (BSIT) and comparative intradermal (CIT) tests in vaccinated (VH) and Testing and culling (TC) herds according to the time elapsed since their individual inclusion in the program. Regarding the BSIT, no significant differences in the proportion of positive reactions was observed at the initial testing between both strategies (p = 1.0000), nor at three (p = 0.2953), four (p = 0.9714) and five (1.0000) years post-vaccination. However, significant differences were observed at one (p<0.0001) and two (p<0.0001) years post-vaccination. These frequencies in the BSIT one and two years after vaccination were significantly higher (p<0.0001) than in any other control in the VH, but from 3 years onwards, differences were not different from the pre-vaccination testing or the other testings (p = 0.5580, p = 0.9931 and p = 1.0000). Frequencies in the TC group were not significantly different from the pre-vaccination frequency at one (p = 0.9941), two (0.9959), three (1.0000), four (p = 1.0000)1 and five (1.0000) years post-vaccination. No statistically significant differences were observed between strategies, or between testings within each strategy in the CIT. Table on the x-axis shows frequencies and table below axis legend shows statistical probabilities for the differences. These p values were obtained with a Student t-test with the Tukey-Kramer adjustment for multiple comparisons on the least square frequency means calculated in the analysis of variance.

As was observed at herd level regarding the BSIT, no significant differences in the proportion of positive reactions was observed at the initial testing between both strategies (p = 1.0000). In the TC herds, no significant change was observed through the subsequent annual testings (Figure 3). However, highly significant differences (p<0.0001) were observed between the initial testing and the following 2 years testings in the VH. When the results in each of the two control strategies were compared regarding the time elapsed since each animal first test (vaccination as replacers), significant differences (p<0.0001) were observed only at the 1 and 2 following yearly testing. From 3 years onwards, differences were not significant even though the proportion still remained slightly higher.

No significant differences were observed when age at vaccination was considered, although slightly lower frequencies of positive results were observed among animals vaccinated less than a year old compared with those vaccinated at older ages (table 1).

**Table 1:** Overall comparison of BSIT and CIT between VH and TC herds in annual testings (least square means and adjusted standard errors).

		Positive animals (%)± Adjusted standard error				
			BSIT		CIT	
**Test & Cull herds (n = 2252)**		**0.5565±0.5100**			**0.0250±0.0772**	
	<12 months (n = 1077)		0.2469±0.7604		0.0500±0.1151	
	> = 12 months (n = 1175)		0.8660±0.6797	p = 0.9299*	0.0000±0.1029	p = 0.9882*
**Vaccinated Herds (n = 2033)**			**9.4180±0.5218**		**0.1026±0.0790**	
	<12 months (n = 939)		9.2989±0.7826		0.0692±0.1185	
	> = 12 months (n = 1094)		9.5371±0.6904	p = 0.9958*	1.3661±0.1045	p = 0.9754*
			p<0.0001**		p = 0.4824**	

Notice that the ratio of positivity in the BSIT test is a highly significant 1/17 between TC and VH animals, while in the CIT it is a non-significant ¼.

*Comparison between vaccinated as calves or cows;

**Comparison between strategies;

BSIT: bovine single intradermal test; CIT: comparative intradermal tests; VH: Vaccinated Herds; TC: Test and Cull herds. Student t-test with the Tukey-Kramer adjustment for multiple comparisons on the least square frequency means calculated in the analysis of variance.

### Slaughterhouse follow-up

None of the slaughtered animals showed any lesion compatible with tuberculosis in the post-mortem inspection carried out by the Public Health Official Veterinary Services.

## Discussion

There are many studies that support the beneficial effect of vaccination for the control of the paratuberculosis as revised by Bastida and Juste [7], however the main hurdle for a wider use of vaccination is still the interference with the diagnosis of tuberculosis with the standard immune skin test. The results shown in this paper indicate that when the BSIT is applied for diagnosis of tuberculosis in vaccinated animals, 6.55% of them would be wrongly diagnosed if the pattern observed here holds for the overall cattle population. However, when the CIT is used, this percentage drops to 0.15%. This results are in agreement with those observed by Chartier *et al.* [11] in paratuberculosis vaccinated goats where, depending on the age, between 2.7% and 50% of the tested animals were reactors to the BSIT when the more stringent test interpretation was applied. In contrast, when making a CIT interpretation, between 0.0% and 2.6% positive reactions were recorded. The usefulness of the comparative test in the diagnosis of tuberculosis in vaccinated animals with whole cell MAP vaccines has been described in different studies in sheep, goats, cattle and deer [12], [13], [14]. Our results also agree with those presented in a recent study carried out in cattle in Denmark with the IFN-γ, where the rate of vaccine cross reactivity was similar (0.7%). However, the use of IFN-γ which is notoriously susceptible to high variability, as well as the fact that it was a post-hoc analysis where the authors acknowledged that large amounts of information were missing [15] might invalidate comparisons. In another study in Spain, using the IFN-γ test on paratuberculosis vaccinated goats, false positive results reached 29% and 21% depending on the cut-off [16]. These results are in sharp contrast with those obtained in goats in France by Chartier *et al.* [11] which were 5.5% and 2.7% at the same cut-offs. The difference seems to be the young age of the Spanish goats that were tested at a median of 17 months of age. Animals in that range of age also showed a 5.5-fold increase in the proportion of positives in the intradermal test of the French study compared with 3.5 years-old adult goats. The time elapsed between vaccination and testing seems to be critical as the results presented here indicate by showing significant differences between the initial testing results and those of the two following years. This significance was lost after 24 months post-vaccination in agreement with the above mentioned results observed by Chartier *et al.* [11]. However, no significant difference was found between samplings when the CIT was used. Even in experimental conditions, the CIT applied to vaccinated calves within their first year after vaccination was 100% specific according to Stabel *et al.* [17].

In our study, the proportion of BSIT positive animals in VH was more than eleven times higher than in the TC herds, but this proportion was reduced to three times with the comparative test. It also could be seen that in the initial pre-vaccination testing (M0) 3.2% of the animals were reactors to the BSIT in both groups. These results indicate that natural infection against paratuberculosis does interfere in the diagnosis of tuberculosis since none of the slaughtered animals showed compatible lesions with tuberculosis in the post-mortem inspection carried out after the slaughtering (data not shown). This interference of natural infection, previously demonstrated by other authors [18], [19], [20], [21], [22], has been estimated to cause between 8.3% and 26.8% false positive reactors using the same techniques and cut-offs that those studies mentioned before. These percentages are similar or even higher than those observed in our vaccinated herds. In spite of this, not all of the authors consider that MAP plays a major role in the appearance of false positive results to tuberculosis in cattle and, on the other hand, periodical culling of paratuberculosis reactors in TC programs is likely to reduce the overall interference rates.

In the current study, the real situation that the Official Veterinary Services responsible of the TB control program found at each yearly control was that, after vaccination, the maximum percentage of BSIT reactors occurred at the M24 testing (20.42%), while the CIT reactors peaked at M12 (0.58%). Taking into account the low number of reactors in the vaccinated herds when the CIT is used, the economic losses due to the disease in the affected farms [2], [23] and the positive effect of the vaccination in the control disease [7] it seems cost-efficient to assume this small lack of specificity of the CIT in the diagnosis of tuberculosis in animals vaccinated against paratuberculosis. That can be considered as irrelevant as it is a very small fraction of the total number of vaccinated animals. Even though paratuberculosis vaccination records were ignored, it would be possible to kill this small number of cases to verify that they are not real TB cases. In our study, only one of these animals had a CIT repeated one year later and then it was clearly negative.

Finally, our results show that there were no significant differences in the proportion of positive results in either the single or the comparative test related to the age of vaccination. However, these results show a certain pattern consisting in that positive results might appear in TC herds at any time in the program, while in the VH these positives would appear mainly in the testings carried out immediately following vaccination.

Another concern raised by paratuberculosis vaccination is the behavior of exposure to *M. bovis* infection of vaccinated animals. Although results in a caprine model show not only that infected animals can be quickly and easily detected, but that a certain degree of cross-protection against tuberculosis can be afforded by paratuberculosis vaccination [24], further research is needed to more precisely clarify this aspect in the bovine species.

### Conclusion

The CIT can be considered an useful tool for which interference caused by vaccination against paratuberculosis in TB free herds included in tuberculosis eradication programs, is not different from that caused by natural infection in non-vaccinated herds. This low level interference does not support, on an evidence-based and cost-efficient decision-making, current reluctance to use vaccination as an economically and epidemiologically highly efficient control tool [3], [25] in affected herds experiencing paratuberculosis production losses.
